# Crystal structure of 4-(2-bromo­prop­ion­yl)-3-phenyl­sydnone

**DOI:** 10.1107/S1600536814022260

**Published:** 2014-10-18

**Authors:** D. Grossie, L. Harrison, K. Turnbull

**Affiliations:** a Wright State University, Chemistry, 3649 Colonel Glenn Hwy, Dayton, Ohio 45435, USA

**Keywords:** crystal structure, sydnone, mesoionic compounds, oxa­diazo­lyliumolate, hydrogen bonding

## Abstract

Sydnones are a class of mesoionic compounds containing a five-membered heterocyclic ring. In general, sydnone com­pounds are synthesized with an aromatic substutuent at the N^3^ position. This feature, adds to the stability of the heterocyclic ring. In the title compound {systematic name: 4-(2-bromo­propano­yl)-3-phenyl-1,2,3λ^5^-oxa­diazol-3-ylium-5-olate}, C_11_H_9_BrN_2_O_3_, the aromatic substitutent is an unsubstituted phenyl ring. The sydnone ring is almost planar, with a maximum deviation from the mean plane of 0.023 (1) Å, but is not coplanar with the phenyl ring, having a dihedral angle of 40.93 (8)°. The carbonyl side chain is twisted relative to the syndone ring by 15.8 (2)°. The mol­ecules are packed in the unit cell as pairs related by an inversion center at (1, 0, 1/2). The pairs inter­act *via* π-stacking, with the distance separating the centroids being 3.824 (1) Å. The Br atom has two contacts, one to an N atom in a neighboring asymmetric unit with a distance of 3.346 (2) Å (the sum of the van der Waals radii is 3.40 Å) and a second to an H atom with a distance of 3.03 Å. The contact with the H atom is perpendicular (C—Br⋯H = 98.60°) to the C—Br bond, and that to the N atom is linear [C—Br⋯N = 169.10 (5)°] to the C—Br bond. The O atom of the sydnone ring is involved in two hydrogen bonds, one intra­molecular with a donor–acceptor distance of 3.1486 (19) Å and a second that is inter­molecular, with a phenyl H atom as the donor and has a donor–acceptor distance of 3.346 (2) Å.

## Related literature   

For more information on the sydnone family of compounds, see: Ohta & Kato (1969 ▶). For synthesis and structural information, see: Hope & Thiessen (1969 ▶); Ollis & Ramsden (1976 ▶); Hodson & Turnbull (1985 ▶); Grossie & Turnbull (1992 ▶); Grossie *et al.* (2001 ▶, 2007 ▶); Riddle *et al.* (2004*a* ▶,*b* ▶,*c* ▶). For further synthesis information, see: Balaguer *et al.* (2013 ▶). For halogen-bond information, see: Politzer *et al.* (2010 ▶).
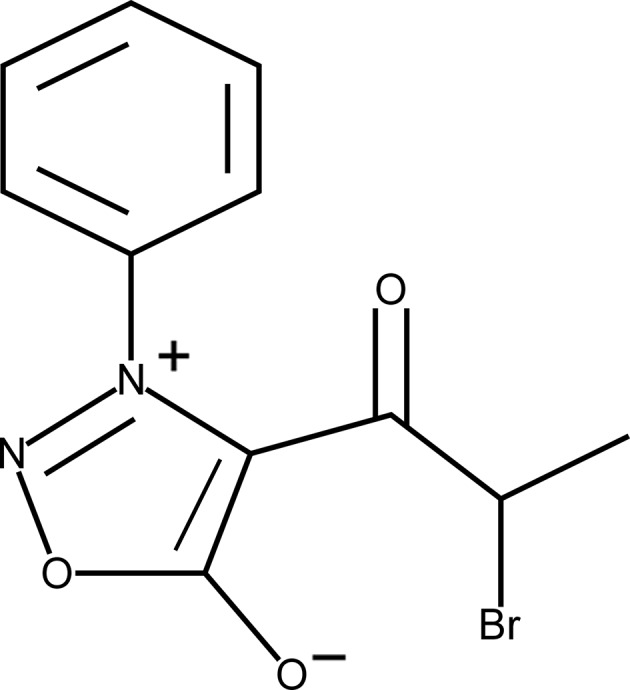


## Experimental   

### Crystal data  

C_11_H_9_BrN_2_O_3_
*M* *_r_* = 297.11
Triclinic, 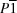

*a* = 7.5388 (8) Å
*b* = 7.8094 (8) Å
*c* = 10.2470 (11) Å
α = 89.1333 (14)°
β = 77.2754 (14)°
γ = 72.6502 (13)°
*V* = 560.86 (10) Å^3^
*Z* = 2
Mo *K*α radiation
μ = 3.66 mm^−1^
*T* = 173 K
0.48 × 0.43 × 0.21 mm

### Data collection  

Bruker APEXII diffractometer
Absorption correction: multi-scan (*SADABS*; Bruker, 2006 ▶) *T*_min_ = 0.586, *T*_max_ = 0.746
8816 measured reflections
3313 independent reflections
3091 reflections with *I* > 2σ(*I*)
*R*_int_ = 0.019

### Refinement  

*R*[*F* ^2^ > 2σ(*F* ^2^)] = 0.025
*wR*(*F* ^2^) = 0.068
*S* = 1.06
3313 reflections
155 parameters
H-atom parameters constrained
Δρ_max_ = 0.83 e Å^−3^
Δρ_min_ = −0.24 e Å^−3^

### 

Data collection: *APEX2* (Bruker, 2006 ▶); cell refinement: *APEX2*; data reduction: *APEX2*; program(s) used to solve structure: *SHELXS97* (Sheldrick, 2008 ▶); program(s) used to refine structure: *SHELXL97* (Sheldrick, 2008 ▶); molecular graphics: *OLEX2* (Dolomanov *et al.*, 2009 ▶); software used to prepare material for publication: *OLEX2*.

## Supplementary Material

Crystal structure: contains datablock(s) I. DOI: 10.1107/S1600536814022260/pk2531sup1.cif

Structure factors: contains datablock(s) I. DOI: 10.1107/S1600536814022260/pk2531Isup2.hkl

Supporting information file. DOI: 10.1107/S1600536814022260/pk2531Isup3.cml

**Fig. 1.**
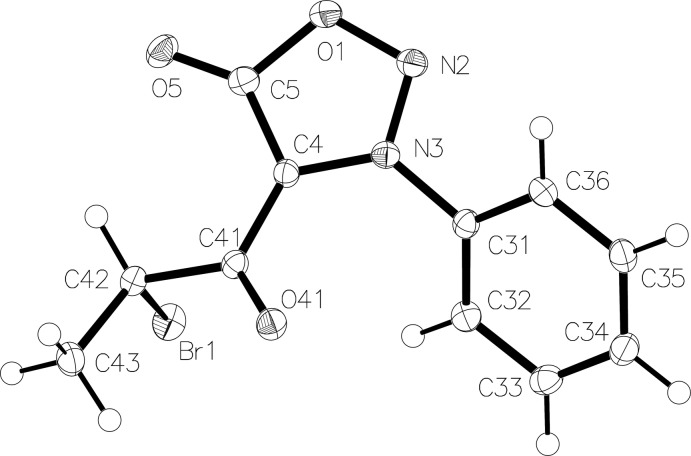
The title compound with displacement ellipsoids drawn at the 50% probability level. H atoms are shown as spheres of arbitary radius.

**Fig. 2.**
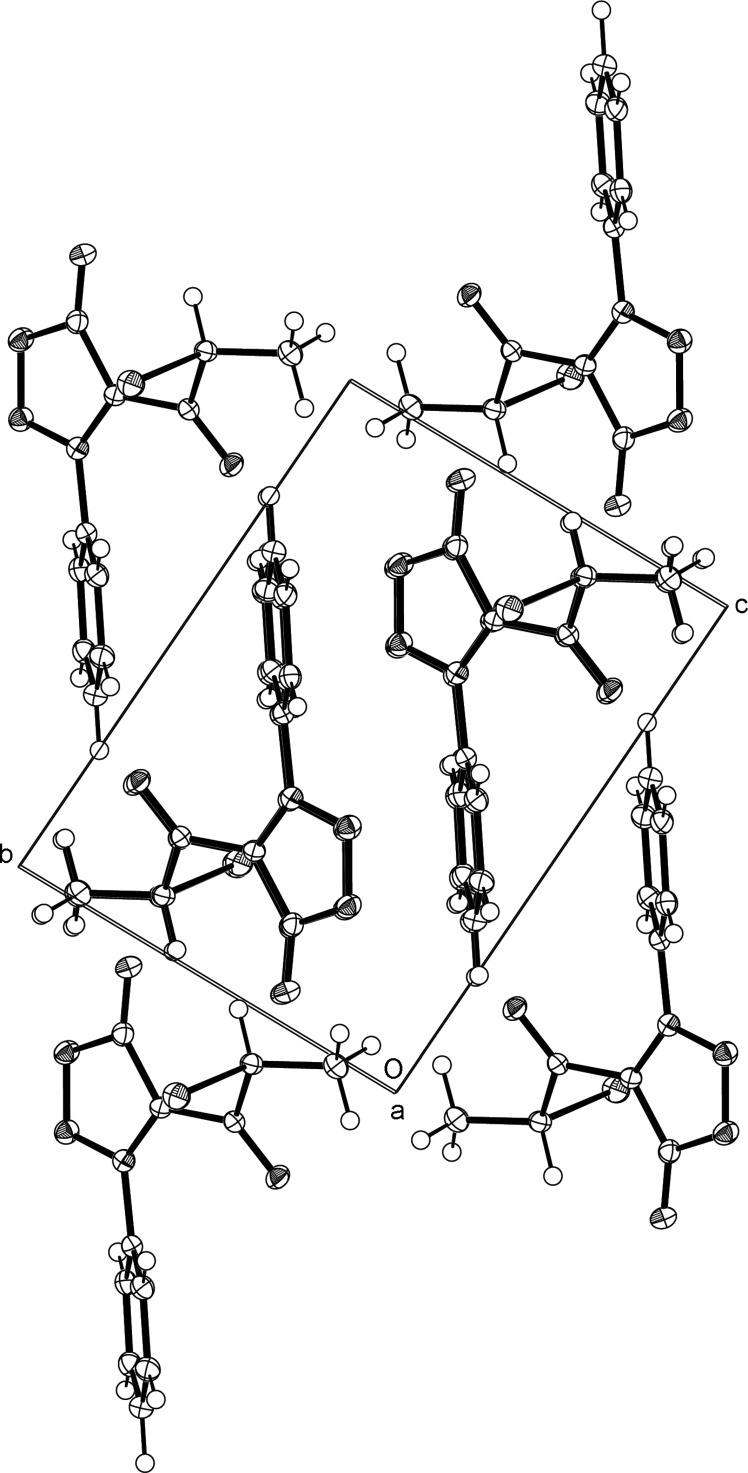
The packing diagram of the title compound, viewed down the a-axis.

. DOI: 10.1107/S1600536814022260/pk2531fig1.tif
The title compound with displacement ellipsoids drawn at the 50% probability level. H atoms are shown as spheres of arbitary radius.

a . DOI: 10.1107/S1600536814022260/pk2531fig2.tif
The packing diagram of the title compound, viewed down the *a*-axis.

CCDC reference: 1028263

Additional supporting information:  crystallographic information; 3D view; checkCIF report

## Figures and Tables

**Table 1:** Hydrogen-bond geometry (, )

*D*H*A*	*D*H	H*A*	*D* *A*	*D*H*A*
C35H35O41^i^	0.95	2.52	3.346(2)	146
C42H42O5	1.00	2.43	3.1486(19)	128

Symmetry code: (i) 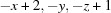.

## supplementary crystallographic information

### S1. Comment

The molecule contains one chiral center, C42, which in the asymmetric unit used in refinement has an S configuration. The sydnone (O1 - C5) and phenyl ring (C31 - C36) are planar with maximum deviation from the mean plane of 0.023 (1) and 0.006 (1) Å, respectively. The angle between the planes of the sydnone (O1 – C5) and phenyl ring (C31 – C36) is 40.93 (8)°. The molecule is packed in the unit cell with the phenyl ring of neighboring molecules lying parallel to one another, with a separation of 3.346 (2) Å. The molecules are related by an inversion center at (1, 0, 0.5), lying head-to-tail with the centroids of the parallel phenyl rings shifted by 1.851 (1) Å. Each pair of molecules is repeated with a separation between nearest rings of 3.317 (2) Å and an offset of 4.600 (2) Å. In addition, weak hydrogen- and halogen-bonding is observed around the bromine atom, a halogen-bond to N2 in a neighboring asymmetric unit with a distance of 3.346 (2) Å and a hydrogen-bond to H42 with a distance of 3.028Å. The hydrogen-bond with H42 has an angle at Br1 of 98.60° and the halogen bond has an angle of 169.10 (5)°. This behavior is characteristic of the "flattening" of halogen atoms in the direction of the C—Br bond as discussed by Politzer *et al.* (2010). The oxygen atom O5 of the sydnone ring is involved in two hydrogen bonds, one intra­molecular to H42 with a D···A distance of 3.1486 (19) Å and a second that is inter­molecular, using a phenyl hydrogen atom, H35, as the donor and has a D···A distance of 3.346 (2) Å. The packing of the molecules is shown in Fig. 2.

### S2. Synthesis

3-Phenyl-4-(2-bromo­propionyl)sydnone was prepared in 84% yield from 3-phenyl-4-propionyl)sydnone (itself synthesized in 63% yield from 3-phenyl­sydnone by Friedel-Crafts acyl­ation with propionic anhydride, bis­muth triflate and lithium perchlorate) by treatment with bromine (10 eq) in glacial acetic acid with a few drops of concentrated sulfuric acid at 40°C for 2 hours (Balaguer *et al.*, 2013).

### S3. Refinement

Crystal data, data collection and structure refinement details are summarized in the following tables. Refinement of the compound resulted in residual positive electron density (0.83 e Å^-3^) in the vicinity the the Br atom. Hydrogen atom positions were calculated using geometric parameters and allowed to refined as a "riding" atom with a isotropic thermal factor equal to 1.5 Ueq of the attached atoms with sp^3^ hybridization and 1.2 Ueq of the attached atoms with sp^2^ hybridization positions.

### Crystal data

**Table:**

C_11_H_9_BrN_2_O_3_	*Z* = 2
*M**_r_* = 297.11	*F*(000) = 296
Triclinic, *P*1	*D*_x_ = 1.759 Mg m^−^^3^
*a* = 7.5388 (8) Å	Mo *K*α radiation, λ = 0.71073 Å
*b* = 7.8094 (8) Å	Cell parameters from 4182 reflections
*c* = 10.2470 (11) Å	θ = 2.7–30.7°
α = 89.1333 (14)°	µ = 3.66 mm^−^^1^
β = 77.2754 (14)°	*T* = 173 K
γ = 72.6502 (13)°	Block, clear colourless
*V* = 560.86 (10) Å^3^	0.48 × 0.43 × 0.21 mm

### Data collection

**Table:**

Bruker APEXII diffractometer	3091 reflections with *I* > 2σ(*I*)
Graphite monochromator	*R*_int_ = 0.019
ω scans	θ_max_ = 31.3°, θ_min_ = 2.0°
Absorption correction: multi-scan (*SADABS*; Bruker, 2006)	*h* = −10→10
*T*_min_ = 0.586, *T*_max_ = 0.746	*k* = −11→11
8816 measured reflections	*l* = −14→14
3313 independent reflections	

### Refinement

**Table:**

Refinement on *F*^2^	Primary atom site location: structure-invariant direct methods
Least-squares matrix: full	Hydrogen site location: inferred from neighbouring sites
*R*[*F*^2^ > 2σ(*F*^2^)] = 0.025	H-atom parameters constrained
*wR*(*F*^2^) = 0.068	*w* = 1/[σ^2^(*F*_o_^2^) + (0.047*P*)^2^ + 0.071*P*] where *P* = (*F*_o_^2^ + 2*F*_c_^2^)/3
*S* = 1.06	(Δ/σ)_max_ = 0.001
3313 reflections	Δρ_max_ = 0.83 e Å^−^^3^
155 parameters	Δρ_min_ = −0.24 e Å^−^^3^
0 restraints	

### Special details

**Table:**

Experimental. Absorption correction: SADABS-2008/1 (Bruker,2008) was used for absorption correction. wR2(int) was 0.1048 before and 0.0276 after correction. The Ratio of minimum to maximum transmission is 0.7846. The λ/2 correction factor is 0.0015.
Geometry. All e.s.d.'s (except the e.s.d. in the dihedral angle between two l.s. planes) are estimated using the full covariance matrix. The cell e.s.d.'s are taken into account individually in the estimation of e.s.d.'s in distances, angles and torsion angles; correlations between e.s.d.'s in cell parameters are only used when they are defined by crystal symmetry. An approximate (isotropic) treatment of cell e.s.d.'s is used for estimating e.s.d.'s involving l.s. planes.Least-squares planes (*x*,*y*,*z* in crystal coordinates) and deviations from them (* indicates atom used to define plane)Sydnone:O1—C50.7220 (5) *x* - 0.4771 (6) *y* + 0.5010 (7) *z* = 7.363 (6)0.023 (1) O1* -0.016 (1) N2* 0.002 (1) N3* 0.012 (1) C4* -0.022 (2) C5*Attached phenyl ring: C31–36-0.1134 (6) *x* + 0.8146 (4) *y* - 0.5688 (5) *z* = -2.369 (5)-0.004 (1) C31* -0.006 (1) C32* 0.004 (1) C33* -0.002 (1) C34* 0.005 (1) C35* -0.005 (1) C36Angle to previous plane (with approximate e.s.d.) = 40.93 (8)

### Fractional atomic coordinates and isotropic or equivalent isotropic displacement parameters (Å^2^)

**Table:**

	*x*	*y*	*z*	*U*_iso_*/*U*_eq_	
Br1	0.08657 (2)	0.41054 (2)	0.79958 (2)	0.02060 (6)	
O5	0.37447 (17)	0.66470 (15)	0.94737 (11)	0.0212 (2)	
O41	0.55129 (15)	0.11464 (14)	0.77220 (11)	0.0188 (2)	
N3	0.66206 (17)	0.42537 (16)	0.66182 (12)	0.0141 (2)	
O1	0.61412 (16)	0.67163 (14)	0.76973 (11)	0.0186 (2)	
N2	0.71705 (18)	0.56946 (17)	0.65504 (13)	0.0174 (2)	
C31	0.7431 (2)	0.30011 (19)	0.54603 (14)	0.0147 (2)	
C4	0.52361 (19)	0.42322 (18)	0.77203 (14)	0.0143 (2)	
C36	0.9357 (2)	0.26502 (19)	0.48800 (15)	0.0163 (3)	
H36	1.0129	0.3173	0.5259	0.020*	
C32	0.6253 (2)	0.2270 (2)	0.49368 (14)	0.0170 (3)	
H32	0.4933	0.2548	0.5346	0.020*	
C33	0.7057 (2)	0.1117 (2)	0.37938 (15)	0.0193 (3)	
H33	0.6287	0.0583	0.3422	0.023*	
C42	0.2688 (2)	0.30934 (19)	0.91455 (14)	0.0153 (3)	
H42	0.2597	0.4037	0.9827	0.018*	
C41	0.4596 (2)	0.26752 (19)	0.81517 (14)	0.0143 (2)	
C5	0.4824 (2)	0.58994 (19)	0.84533 (15)	0.0164 (3)	
C34	0.8990 (2)	0.0748 (2)	0.31958 (15)	0.0202 (3)	
H34	0.9531	−0.0035	0.2415	0.024*	
C35	1.0130 (2)	0.1516 (2)	0.37324 (15)	0.0186 (3)	
H35	1.1444	0.1264	0.3313	0.022*	
C43	0.2287 (2)	0.1487 (2)	0.98465 (15)	0.0206 (3)	
H43A	0.0956	0.1825	1.0349	0.031*	
H43B	0.3133	0.1080	1.0465	0.031*	
H43C	0.2511	0.0514	0.9180	0.031*	

### Atomic displacement parameters (Å^2^)

**Table:**

	*U*^11^	*U*^22^	*U*^33^	*U*^12^	*U*^13^	*U*^23^
Br1	0.01601 (8)	0.02299 (9)	0.02065 (9)	−0.00160 (6)	−0.00565 (5)	0.00083 (6)
O5	0.0253 (6)	0.0159 (5)	0.0184 (5)	−0.0025 (4)	−0.0019 (4)	−0.0031 (4)
O41	0.0191 (5)	0.0133 (5)	0.0201 (5)	−0.0018 (4)	−0.0003 (4)	−0.0004 (4)
N3	0.0130 (5)	0.0146 (5)	0.0152 (5)	−0.0042 (4)	−0.0042 (4)	0.0009 (4)
O1	0.0204 (5)	0.0160 (5)	0.0201 (5)	−0.0069 (4)	−0.0040 (4)	−0.0013 (4)
N2	0.0176 (6)	0.0168 (5)	0.0188 (6)	−0.0068 (4)	−0.0039 (4)	0.0004 (4)
C31	0.0161 (6)	0.0142 (6)	0.0131 (6)	−0.0038 (5)	−0.0032 (5)	0.0015 (5)
C4	0.0142 (6)	0.0142 (6)	0.0134 (6)	−0.0038 (5)	−0.0015 (5)	0.0003 (5)
C36	0.0162 (6)	0.0163 (6)	0.0167 (6)	−0.0060 (5)	−0.0033 (5)	0.0030 (5)
C32	0.0163 (6)	0.0189 (6)	0.0165 (6)	−0.0053 (5)	−0.0051 (5)	0.0021 (5)
C33	0.0241 (7)	0.0191 (7)	0.0172 (6)	−0.0076 (5)	−0.0083 (5)	0.0012 (5)
C42	0.0159 (6)	0.0158 (6)	0.0131 (6)	−0.0035 (5)	−0.0025 (5)	−0.0001 (5)
C41	0.0159 (6)	0.0144 (6)	0.0124 (6)	−0.0038 (5)	−0.0039 (5)	0.0013 (5)
C5	0.0186 (6)	0.0144 (6)	0.0168 (6)	−0.0043 (5)	−0.0059 (5)	0.0018 (5)
C34	0.0263 (7)	0.0170 (6)	0.0153 (6)	−0.0054 (5)	−0.0021 (5)	0.0001 (5)
C35	0.0186 (6)	0.0170 (6)	0.0175 (6)	−0.0043 (5)	−0.0002 (5)	0.0025 (5)
C43	0.0231 (7)	0.0206 (7)	0.0168 (7)	−0.0078 (6)	−0.0003 (5)	0.0024 (5)

### Geometric parameters (Å, º)

**Table:**

Br1—C42	1.9842 (14)	C32—H32	0.9500
O5—C5	1.2070 (18)	C32—C33	1.394 (2)
O41—C41	1.2192 (17)	C33—H33	0.9500
N3—N2	1.3062 (17)	C33—C34	1.394 (2)
N3—C31	1.4483 (18)	C42—H42	1.0000
N3—C4	1.3622 (18)	C42—C41	1.5158 (19)
O1—N2	1.3701 (16)	C42—C43	1.512 (2)
O1—C5	1.4200 (18)	C34—H34	0.9500
C31—C36	1.3873 (19)	C34—C35	1.388 (2)
C31—C32	1.3872 (19)	C35—H35	0.9500
C4—C41	1.4649 (19)	C43—H43A	0.9800
C4—C5	1.4289 (19)	C43—H43B	0.9800
C36—H36	0.9500	C43—H43C	0.9800
C36—C35	1.389 (2)		
			
N2—N3—C31	115.61 (12)	C41—C42—H42	109.5
N2—N3—C4	114.72 (12)	C43—C42—Br1	111.26 (10)
C4—N3—C31	129.37 (12)	C43—C42—H42	109.5
N2—O1—C5	110.83 (10)	C43—C42—C41	114.62 (12)
N3—N2—O1	105.26 (11)	O41—C41—C4	122.48 (13)
C36—C31—N3	117.95 (13)	O41—C41—C42	121.98 (13)
C32—C31—N3	119.37 (12)	C4—C41—C42	115.52 (12)
C32—C31—C36	122.61 (13)	O5—C5—O1	120.00 (13)
N3—C4—C41	125.59 (12)	O5—C5—C4	136.41 (14)
N3—C4—C5	105.46 (12)	O1—C5—C4	103.57 (12)
C5—C4—C41	128.09 (13)	C33—C34—H34	119.7
C31—C36—H36	120.8	C35—C34—C33	120.51 (14)
C31—C36—C35	118.43 (14)	C35—C34—H34	119.7
C35—C36—H36	120.8	C36—C35—H35	119.9
C31—C32—H32	120.9	C34—C35—C36	120.20 (13)
C31—C32—C33	118.20 (13)	C34—C35—H35	119.9
C33—C32—H32	120.9	C42—C43—H43A	109.5
C32—C33—H33	120.0	C42—C43—H43B	109.5
C32—C33—C34	120.04 (14)	C42—C43—H43C	109.5
C34—C33—H33	120.0	H43A—C43—H43B	109.5
Br1—C42—H42	109.5	H43A—C43—H43C	109.5
C41—C42—Br1	102.16 (9)	H43B—C43—H43C	109.5
			
Br1—C42—C41—O41	−103.92 (14)	C31—C36—C35—C34	−0.5 (2)
Br1—C42—C41—C4	74.62 (12)	C31—C32—C33—C34	−1.0 (2)
N3—C31—C36—C35	−177.06 (13)	C4—N3—N2—O1	1.77 (16)
N3—C31—C32—C33	177.78 (13)	C4—N3—C31—C36	−144.40 (15)
N3—C4—C41—O41	15.8 (2)	C4—N3—C31—C32	38.7 (2)
N3—C4—C41—C42	−162.76 (13)	C36—C31—C32—C33	1.0 (2)
N3—C4—C5—O5	178.85 (18)	C32—C31—C36—C35	−0.2 (2)
N3—C4—C5—O1	−2.85 (15)	C32—C33—C34—C35	0.3 (2)
N2—N3—C31—C36	42.21 (18)	C33—C34—C35—C36	0.5 (2)
N2—N3—C31—C32	−134.71 (14)	C41—C4—C5—O5	−11.4 (3)
N2—N3—C4—C41	−169.29 (13)	C41—C4—C5—O1	166.87 (13)
N2—N3—C4—C5	0.77 (17)	C5—O1—N2—N3	−3.68 (15)
N2—O1—C5—O5	−177.26 (13)	C5—C4—C41—O41	−152.03 (15)
N2—O1—C5—C4	4.09 (15)	C5—C4—C41—C42	29.4 (2)
C31—N3—N2—O1	176.15 (11)	C43—C42—C41—O41	16.5 (2)
C31—N3—C4—C41	17.3 (2)	C43—C42—C41—C4	−164.94 (13)
C31—N3—C4—C5	−172.67 (13)		

### Hydrogen-bond geometry (Å, º)

**Table:**

*D*—H···*A*	*D*—H	H···*A*	*D*···*A*	*D*—H···*A*
C35—H35···O41^i^	0.95	2.52	3.346 (2)	146
C42—H42···O5	1.00	2.43	3.1486 (19)	128

Symmetry code: (i) −*x*+2, −*y*, −*z*+1.

### Halogen-bond Geometry (Å, °)

**Table:**

D-Br···A	D-Br	Br···A	D···A	D-Br···A
C42-Br1···N2^i^	1.9842 (15)	3.3458 (14)	5.307 (2)	169.10 (5)
C42-Br1···H42^ii^	1.9842 (15)	3.03	3.860	98.60

(i) 1-x,y,z (ii) -x,1-y,2-z
