# Supplementary figures and images for: Crystal structure of 4-(2-bromo­prop­ion­yl)-3-phenyl­sydnone
Source: Acta Crystallogr Sect E Struct Rep Online. 2014 Oct 18;70(Pt 11):o1165–6. doi: 10.1107/S1600536814022260 (PMC4257336; doi:10.1107/S1600536814022260)

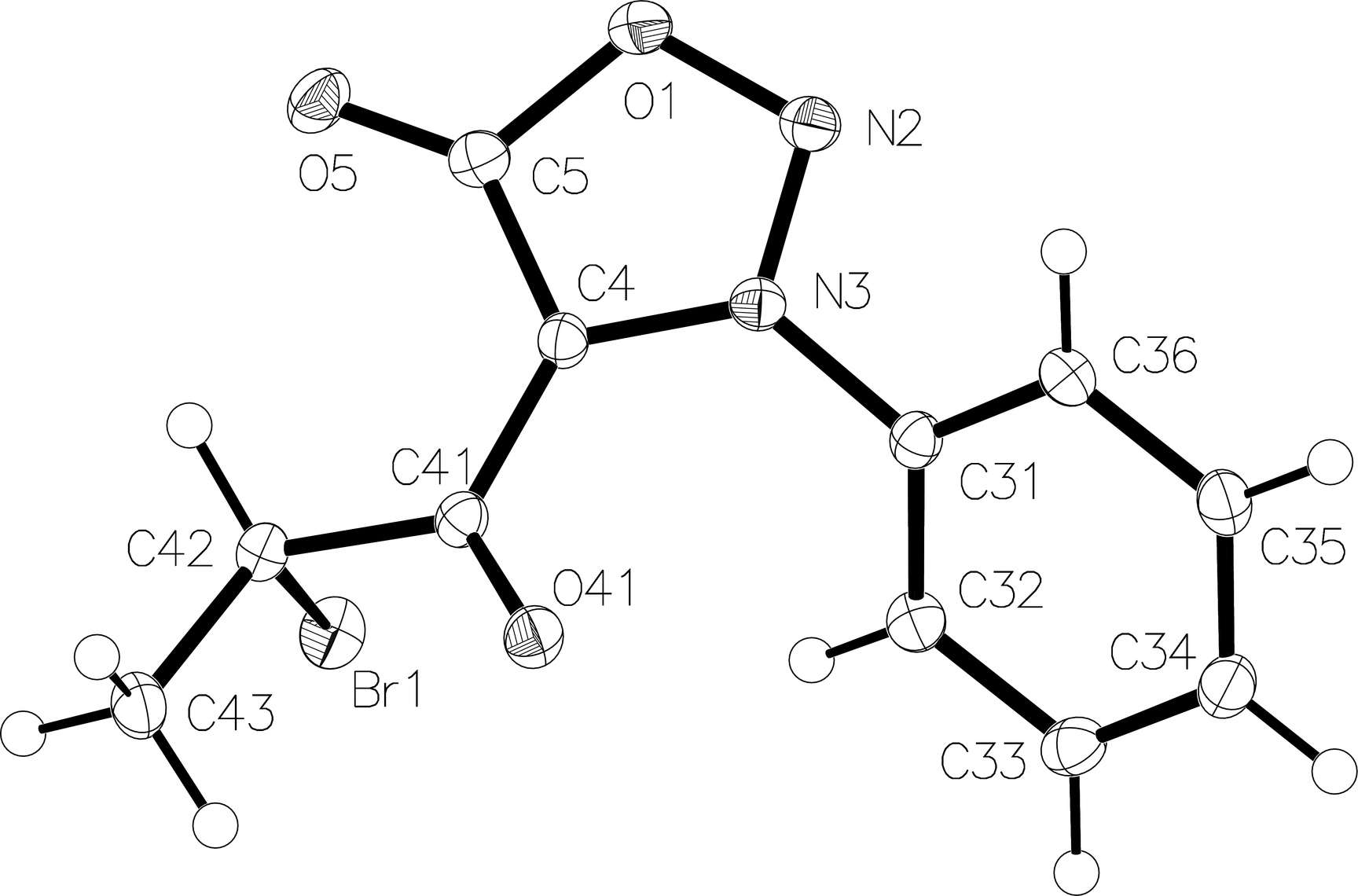

Supplement: Supplementary file 4 [file e-70-o1165-fig1.tif]

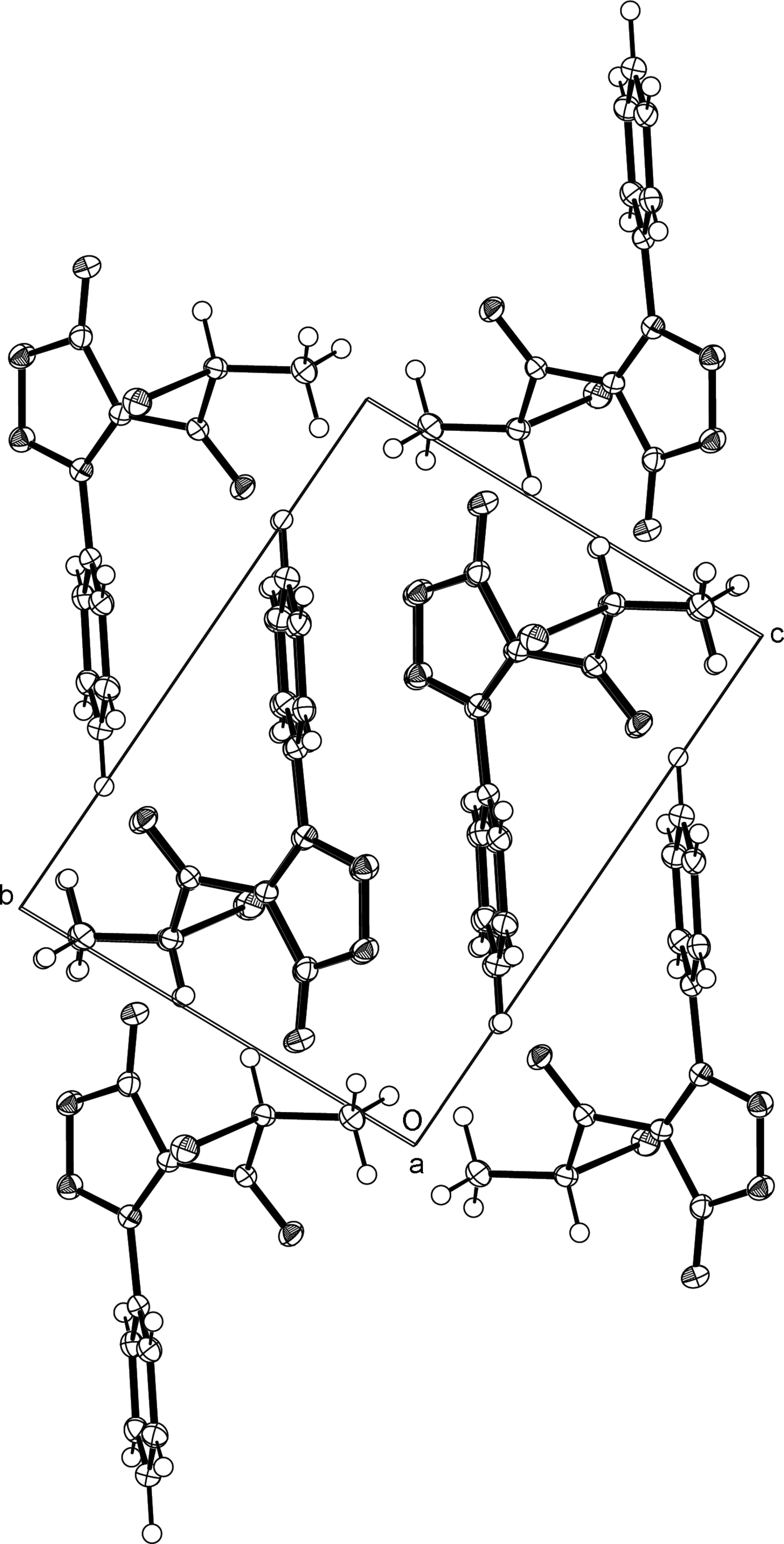

Supplement: Supplementary file 5 [file e-70-o1165-fig2.tif]
